# Genome-wide association study to reveal new candidate genes using single-step approaches for productive traits of Yorkshire pig in Korea

**DOI:** 10.5713/ab.23.0255

**Published:** 2024-01-20

**Authors:** Jun Park

**Affiliations:** 1 Department of Animal Biotechnology, Jeonbuk National University, Jeonju 54896, Korea

**Keywords:** Gene Ontology, Genome-wide Association Study, Kyoto Encyclopedia of Genes and Genomes, Productive Traits, Yorkshire Pig

## Abstract

**Objective:**

The objective is to identify genomic regions and candidate genes associated with age to 105 kg (AGE), average daily gain (ADG), backfat thickness (BF), and eye muscle area (EMA) in Yorkshire pig.

**Methods:**

This study used a total of 104,380 records and 11,854 single nucleotide polymorphism (SNP) data obtained from Illumina porcine 60K chip. The estimated genomic breeding values (GEBVs) and SNP effects were estimated by single-step genomic best linear unbiased prediction (ssGBLUP).

**Results:**

The heritabilities of AGE, ADG, BF, and EMA were 0.50, 0.49, 0.49, and 0.23, respectively. We identified significant SNP markers surpassing the Bonferroni correction threshold (1.68×10^−6^), with a total of 9 markers associated with both AGE and ADG, and 4 markers associated with BF and EMA. Genome-wide association study (GWAS) analyses revealed notable chromosomal regions linked to AGE and ADG on *Sus scrofa* chromosome (SSC) 1, 6, 8, and 16; BF on SSC 2, 5, and 8; and EMA on SSC 1. Additionally, we observed strong linkage disequilibrium on SSC 1. Finally, we performed enrichment analyses using gene ontology and Kyoto encyclopedia of genes and genomes (KEGG), which revealed significant enrichments in eight biological processes, one cellular component, one molecular function, and one KEGG pathway.

**Conclusion:**

The identified SNP markers for productive traits are expected to provide valuable information for genetic improvement as an understanding of their expression.

## INTRODUCTION

Over time, genetic improvement in pig breeding for economically important traits has been achieved through continuous research efforts. Productive traits, including average daily gain (ADG), age to 105 kg (AGE), backfat thickness (BF), and eye muscle area (EMA) exhibit moderate to high heritability, enabling their enhancement through selective breeding strategies [1]. ADG and AGE directly impact pig growth [2,3], while BF plays a crucial role in the reproductive performance of Landrace and Yorkshire sows [4], thereby influencing the breeding potential of the maternal line.

According to the Korean Swine Performance Recording Standards (KSPRS) established by the Ministry of Agriculture, Food and Rural Affairs (MAFRA), performance testing is conducted within the weight range of 70 to 110 kg, with the current endpoint set at 90 kg. To assess growth trait performance, the number of days required to reach 90 kg and backfat thickness are considered. However, the 90 kg endpoint weight has remained unchanged since its establishment in 1984, reflecting the market weight of finishing pigs at that time. Considering the prevailing trend of market weights exceeding 110 kg, there is a consensus emerging that the endpoint weight for performance testing should be adjusted accordingly. Consequently, a new adjustment formula based on a weight of 105 kg has been developed by the National Institute of Animal Science (NIAS). This updated formula aligns more closely with market weights, enabling accurate evaluation of productive traits in breeding animals and enhancing genetic improvement and efficiency.

Genome-wide association study (GWAS) have been extensively applied to various domains, including the identification of genetic variants associated with economically significant traits. Most economic traits in livestock exhibit quantitative nature with polygenic inheritance patterns, thereby making their underlying genetic mechanisms not fully elucidated. Multiple candidate genes and significant markers have been reported for the same trait, often showing associations between multiple traits at the same genomic locus. While these findings are inherent to quantitative traits, GWAS by single marker analysis may have limited power in detecting quantitative trait loci (QTLs) and mapping accuracy [5]. Moreover, the cost associated with analyzing single nucleotide polymorphism (SNP) panels and the availability of genomic data across individuals pose additional challenges. Several recent studies have employed this approach to explore production, carcass, and reproductive traits in livestock species [6–8].

In this study, our objective is using single-step approaches to identify genomic regions and candidate genes associated with productive traits (AGE, ADG, BF, and EMA) in Yorkshire pig. Additionally, we conducted gene ontology (GO) and Kyoto encyclopedia of genes and genomes (KEGG) enrichment analyses to gain deeper insights into the underlying biological processes and functional terms associated with the identified candidate genes for productive traits.

## MATERIALS AND METHODS

### Animals and phenotypes

The animals used in this study were raised in five great-grand-parents farms in Korea. In brief, a total of 104,380 Yorkshire (17,899 males and 86,481 females) born between 2015 and 2021 were used in this study (Supplementary Table S1). In this study, productive traits such as AGE, ADG, BF, and EMA adjusted to 105 kg were calculated as reported by the NIAS in Korea (https://www.nias.go.kr/images/promote/result/file/2021_2_5.pdf).

### Single nucleotide polymorphism data and quality control

In this study, the Illumina Porcine 60K V1 and V2 were used, and V2 was selected as the reference panel for imputation. Prior to imputation, phasing was performed using Shapeit4 [9], which is a fast and accurate method for haplotype estimation that uses a PBWT-based approach to select informative conditioning haplotypes. Imputation was then conducted using Impute5 [10], which assumes phased samples with no missing alleles. After imputation, quality control (QC) was performed by PLINK v1.90 [11] to exclude SNPs with low call rates (<90%), low minor allele frequencies (<0.01), or deviation from Hardy-Weinberg equilibrium (10^−6^). After QC, we used the number of animals and SNPs were 11,854 and 29,732, respectively.

### Statistical analysis

We estimated the genetic parameters of AGE, ADG, BF, and EMA by average information restricted maximum likelihood method. We considered two approaches: pedigree-based best linear unbiased prediction (PBLUP) and single-step genomic best linear unbiased prediction (ssGBLUP). Each trait was estimated with a single-trait animal model, and the equation as follows:


y=Xb+Za+e

where *y* is the vector of phenotypic observations; *b* is the vector of fixed effects (herd-birth year-season, sex); *a* is the vector of additive genetic effects; *e* is the vector of residuals; and *X* and *Z* are the incidence matrices for *b*, *a*, and *e*. Heritability was estimated as $h^{2}=\frac{\sigma_{a}^{2}}{\sigma_{a}^{2}+\sigma_{e}^{2}}$, where $\sigma_{a}^{2}$ and $\sigma_{e}^{2}$ were additive genetic and residual variances, respectively.

Furthermore, GEBVs calculated using ssGBLUP approach, and marker effects were derived from these GEBVs. In contrast to the conventional BLUP approach, ssGBLUP substituted the inverse of the pedigree relationship matrix (*A*^−1^) with the inverse of the combined matrix *H*^−1^, which incorporated both the pedigree and genomic relationship matrices [12]. The *H*^−1^ can be represented as follows:


$$H^{-1}=A^{-1}+\left[\begin{array}{cc} 0 & 0\\ 0 & G^{-1}-A_{22}^{-1} \end{array}\right]$$

where *A* is the numerator relationship matrix based on pedigree for all animals; *A*_22_ is the numerator relationship matrix for genotyped animals; and *G* is the genomic relationship matrix [13]. The SNP effect of each marker was estimated using the reverse operation method of GEBVs, using the following equation:


$$\hat{u}=\lambda DM^{\prime }G^{*-1}\hat{a_{g}}=DM^{\prime }{\left(MDM\right)}^{-1}\hat{a_{g}}$$

where, *û* is the vector of the SNP effect, $\hat{a_{g}}$ is the vector of the GEBV, *M* is the coefficient matrix of the SNP, and *D* is a weighted vector [14]. This study utilized the BLUPF90 software family [15], which includes RENUMF90, BLUPF90+, and POSTGSF90, for the GWAS. The p-values were used to generate a Manhattan plot using the R software and CMplot package [16,17].

### Linkage analyses

In this study, we conducted linkage analyses between the most significant regions associated with teat traits and the identified SNPs within these regions. To investigate the distribution of linkage disequilibrium (LD) blocks in the genotype data after completing SNP QC, we utilized the Haploview software, which generates marker quality statistics, LD information, haplotype blocks, population haplotype frequencies, and single marker association statistics in a user-friendly format [18]. Haploview calculates various pairwise measures of LD and utilizes them to create a graphical representation. Thus, we performed LD block analysis and visualization of the identified SNPs on SSC 1 in the three pig breeds. The visualization using Haploview represents LD values between markers with colors, with stronger associations displayed in shades of red.

### Identification of candidate genes and functional enrichment analysis

We conducted gene annotation for the markers showing significance in the GWAS analysis. The significance level was determined using the Bonferroni suggestive threshold, and markers surpassing this threshold were subjected to gene annotation and functional enrichment analysis. To identify genes within the identified QTL regions, particularly within the significant windows, we utilized the ensemble *Sus scrofa* 11.1 database (https://www.ensembl.org/biomart). Furthermore, to gain deeper insights into the biological processes associated with these regions, we performed GO and KEGG analyses using the Database for Annotation, Visualization, and Integrated Discovery (DAVID v6.8, https://david.ncifcrf.gov/). GO terms and KEGG pathways showing significant enrichment were determined based on a p-value threshold of <0.05. Through these analyses, we gained valuable knowledge regarding crucial molecular pathways and biological functions associated with the observed genetic variations.

## RESULTS AND DISCUSSION

### Heritability

We compared the heritability of productive traits obtained between PBLUP and ssGBLUP (Table 1). Except for EMA, all other traits exhibited heritability values of 0.45 or higher. Comparing PBLUP and ssGBLUP, the estimated heritability values were consistently higher with ssGBLUP. The ssGBLUP method, which incorporates both pedigree and genotypic information, theoretically provides more accurate estimates of genetic parameters [6].

### Genome-wide association study and linkage disequilibrium analysis

**Figure 1:**
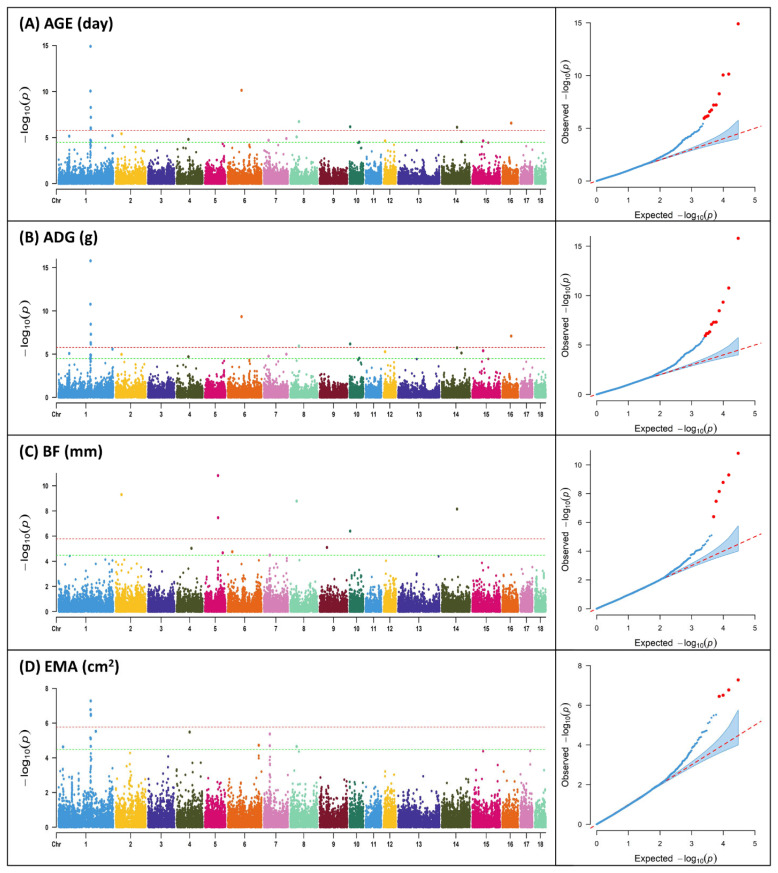
Manhattan plots and quantile – quantile (Q-Q) plot of genome-wide association analysis for productive traits; (A) age to 105 kg (AGE); (B) average daily gain (ADG); (C) backfat thickness (BF); (D) eye muscle area (EMA). Y-axis represents −log_10_ of p-values and X-axis represents chromosome number. The red horizontal line indicates the Bonferroni significance threshold 1.68×10^−6^. The green horizontal line indicates the Bonferroni suggestive threshold 3.36×10^−5^.

In the majority of instances, livestock's primary economic characteristics comprise quantitative traits, with the exception of select specific traits. These quantitative traits possess an intricate genetic architecture, rendering the identification of candidate genes a paramount goal in animal breeding programs. These traits represent pivotal determinants that significantly influence the financial gains of agricultural enterprises, thus necessitating their careful consideration during breeding assessments. In this study, SNP markers showing significance above the Bonferroni correction (1.68×10^−6^) were found to be identical for AGE and ADG, with a total of 9 markers, while for BF and EMA, 4 markers were identified (Table 2; Figure 1). The region with the highest number of markers detected, excluding BF, was found on SSC 1 for all three traits, and for BF, two markers were found on SSC 5. Previous GWAS studies on productive traits adjusted to 100 kg in Duroc, Landrace, Yorkshire, and Pietrain pig populations reported significant SNPs associated with ADG on SSC 1, 3, 7, 10, and 13, and significant SNPs associated with AGE on SSC 1, 3, 4, 6, 7, 8, 9, and 13 [19].

In our study, the significant regions identified through GWAS for AGE and ADG were consistently the same. The most significant marker for both traits was ALGA0006623, which is located in the 5' untranslated region region of the gene *ENSSSCG00000048538*. ALGA0006623 has been reported as a significant marker and gene associated with lean meat percentage (LMP) and ADG in American Duroc and Canadian Duroc pigs [20]. Furthermore, it was identified as the most significant SNP in relation to the fatness trait in pigs [21]. The marker ASGA0038525 was found to be common for AGE, BF, and EMA, with significance above the Bonferroni threshold observed for BF. For complex quantitative traits, it is often more appropriate to assume a nonlinear relationship of gene effects rather than a linear one [19], as genes may contribute differently, and pleiotropic effects of QTLs between traits can occur [5]. Pleiotropic QTLs are common in the pig genome, as exemplified by QTLs related to vertebral number, body length, and nipple number located on SSC 7 [22]. The ASGA0038525 marker identified in our study can be considered as an example of such pleiotropic effects on quantitative traits. Additionally, the 6 markers found on SSC 1 can be regarded as having pleiotropic effects on AGE, ADG, and EMA.

**Figure 2:**
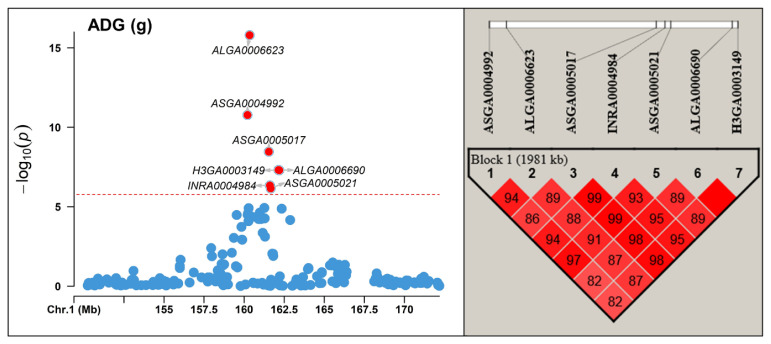
Linkage disequilibrium (LD) block analysis between significant markers found *Sus scrofa* chromosome (SSC) 1 region using Haploview for average daily gain (ADG). The red horizontal line indicates the Bonferroni significance threshold 1.68×10^−6^.

In this study, we observed strong LD among significant markers identified on SSC 1 for AGE, ADG, and EMA, as confirmed by the LD analysis using Haploview (Figure 2; Supplementary Figure S1). Specifically, we detected LD between the SNP ASGA0005017 located within the *CCBE1* gene and adjacent SNPs, spanning a 1.9 Mb region for AGE and ADG, and a 1.8 Mb region for EMA. Within these regions, the widely known *MC4R* gene, associated with pig growth, fat deposition, and feed intake [23,24], is located, indicating its potential as a candidate gene for the observed LD and significant effects within this region. Therefore, if *MC4R* is considered a causal gene, this supports the presence of LD and suggests that the observed significant effects in this region may be influenced by the observed LD.

According to a GWAS study on feed efficiency and related traits in Yorkshire and Duroc pigs, the markers ASGA0004992, ASGA0005017, and ALGA0006707, located on SSC 1, were reported as markers associated with ADG in Yorkshire pigs in the PigQTLdb [25]. The marker ALGA0006707, located near the *MC4R* gene, is widely known as a gene that significantly influences pig growth traits and average feed intake [23,26–28]. The *ALPK2* gene on SSC 1 plays important roles in cardiogenesis and is specifically expressed in muscle tissue, including the longissimus dorsi muscle, where it was upregulated in Wannanhua pigs compared to Yorkshire pigs [29,30]. The *ABL1* gene has been reported as a gene associated with fat metabolism in Yorkshire pigs [31] and considered as a candidate gene related to meat-to-fat ratio [32]. The *CPE* gene is hypothesized to be a candidate gene for meat quality traits related to intramuscular fat level and glucose metabolism in bovines [33].

The *CCND2* gene's expression is significantly associated with the lead SNP in the liver, lung, and spleen, and it has been reported as a gene that influences backfat thickness [34]. *CCND2*, a candidate gene for back quality in Landrace pigs, is also essential for the growth of pancreatic islets, which regulate animal growth through hormonal activities [35]. Recent research identified *CCND2* as the most likely causal gene for backfat thickness and osteochondrosis in pigs [36]. The *APBB2* gene, located on SSC 8, may have roles in cellular and physiological functions related to BF accumulation [37].

In this study, we compared the markers and candidate genes showing significance for each growth trait with previous research findings. While some of the identified markers and candidate genes were consistent with traits reported in previous studies, we also found instances where candidate genes associated with ADG showed significance for other traits such as BF or LMP in other studies. This suggests that economic traits in pigs, being quantitative traits, can exhibit pleiotropic effects. Furthermore, the involvement of genetic associations between productive traits can also account for these results. Moreover, the research findings specifically related to EMA in pigs were sparse, and no reports on the candidate genes identified through GWAS were found. However, we observed that the markers showing significance for EMA in this study were mostly overlapping with the markers identified for ADG and AGE, and they also included markers found for BF. Therefore, the markers and candidate genes identified in this study provide valuable information for understanding the complex genetic influences underlying quantitative traits.

### Gene ontology terms and Kyoto encyclopedia of genes and genomes pathway enrichment analysis

In this study, gene set enrichment analyses were conducted to examine the associations between various terms and productive traits. The results revealed significant enrichments in 8 biological processes, 1 cellular component, 1 molecular function, and 1 KEGG pathway. Among them, the most significant GO term identified was GO:0002020, which pertains to protease binding (Table 3). Although the GO terms associated with the *CCDH20* and *CDH8* genes were frequently observed, there have been no reports on their association with productive traits in pigs and other livestock.

Positive regulation of interleukin-2 production (GO:003 2743) refers to any process that activates or increases the frequency, rate, or extent of interleukin-2 production. One of the genes related to this term, *MAP3K7*, has been reported to be associated with growth traits [38]. Positive regulation of bone resorption (GO:0045780) refers to any process that activates or increases the frequency, rate, or extent of bone resorption. *FSHB*, one of the genes associated with this term, has been reported to be linked to the major gene controlling litter size [39], and it also affects reproductive capacity [40].

Phosphatidylinositol phosphate biosynthetic process (GO: 0046854) refers to the chemical reactions and pathways that result in the formation of phosphatidylinositol phosphate. Phosphatidylinositol is involved in various physiological functions in the body, including muscle contraction, cell proliferation, and differentiation [41]. Furthermore, one of the genes associated with this term, *IP6K3*, has recently been announced as a candidate gene related to body weight [5]. *IP6K3* encodes inositol hexakisphosphate kinase 3, which generates inositol pyrophosphates and regulates diverse cellular functions, including metabolism and body weight. Mice lacking this gene exhibited lower growth rates, impaired metabolism, and shorter lifespans [42].

The *SOCS2* gene is one of the genes associated with cell growth, metabolism, and immunity [43]. It has been reported as a gene that prevents the occurrence of various tumors and liver diseases caused by fat accumulation [44,45]. The results of the GO enrichment analyses further support the involvement of numerous genes in growth development.

## CONCLUSION

The identified SNP markers for productive traits are expected to provide valuable information for genetic improvement as an understanding of their expression. In this study, we have identified novel genes (ENSSSCG00000052156, ENSSSCG 00000033497, ENSSSCG00000046071) associated with productive traits in Yorkshire pigs. Furthermore, the GO and KEGG analyses revealed that, except for GO:0032743 and GO:0042981, the remaining GO terms and KEGG pathways have not been directly implicated in the study of productive traits in Yorkshire pigs. With the accumulation of more phenotype and SNP data in the future, it is anticipated that more effective SNP markers can be identified.

## Figures and Tables

**Table 1:** Variance components and heritabilities for productive traits

Traits	Method	$\sigma_{a}^{2}$	$\sigma_{e}^{2}$	$\sigma_{P}^{2}$	*h*^2^ (SE)
AGE (d)	PBLUP	59.24	69.29	128.53	0.46 (0.01)
	ssGBLUP	66.74	67.31	134.04	0.50 (0.01)
ADG (g)	PBLUP	1,029.10	1,248.80	2,278.00	0.45 (0.01)
	ssGBLUP	1,163.50	1,211.60	2,375.00	0.49 (0.01)
BF (mm)	PBLUP	3.28	3.93	7.21	0.45 (0.01)
	ssGBLUP	3.70	3.81	7.51	0.49 (0.01)
EMA (cm^2^)	PLBUP	1.93	6.60	8.52	0.22 (0.01)
	ssGBLUP	2.01	6.62	8.63	0.23 (0.01)

$\sigma_{a}^{2}$, additive genetic, $\sigma_{e}^{2}$, residual, $\sigma_{P}^{2}$, phenotypic variances, *h*^2^ (SE), heritability and standard error; PBLUP, pedigree-based best linear unbiased prediction; ssGBLUP, single-step genomic best linear unbiased prediction; AGE, age to 105 kg; ADG, average daily gain; BF, backfat thickness; EMA, eye muscle area.

**Table 2:** Significant SNPs associated with productive traits and their annotated candidate genes

Traits	SNP	SSC	Position	p-value^1)^	Variant	Gene annotation^2)^
AGE (d)	ALGA0006623	1	160347188	1.26E-15	5_prime_UTR	*ENSSSCG00000048538*
	ASGA0004992	1	160210902	8.97E-11	Intergenic	*CDH20 (293757), ENSSSCG00000048538 (130158)*
	ASGA0005017	1	161540913	5.45E-09	Intron	*CCBE1*
	ALGA0006690	1	162147201	6.19E-08	Intergenic	*MALT1 (2321), ALPK2 (28372)*
	H3GA0003149	1	162192627	6.36E-08	Intron	*ALPK2*
	ASGA0005021	1	161657225	1.13E-06	Intron	*CPLX4*
	ALGA0112558	1	270826950	6.37E-06	Intron	*ABL1*
	MARC0037204	1	53549421	7.03E-06	Intergenic	*CEP162 (107022), TBX18 (288768)*
	ALGA0006707	1	162345362	2.34E-05	Intron	*ENSSSCG00000052156*
	ALGA0012897	2	30421810	3.85E-06	Intergenic	*FSHB (22536), KCNA4 (183754)*
	DRGA0004818	4	59203000	1.58E-05	Intergenic	*PKIA (1225426), PEX2 (49337)*
	ALGA0105098	6	69291261	7.32E-11	Intron	*ENSSSCG00000033497*
	H3GA0023150	7	113231192	1.30E-05	Intron	*CATSPERB*
	ALGA0047819	8	43646405	1.84E-07	Intron	*CPE*
	ASGA0038525	8	32008618	8.44E-06	Intron	*APBB2*
	ALGA0113142	10	48069639	3.11E-05	Intron	*PRPF18*
	ALGA0064467	12	7009388	2.27E-05	Intergenic	*RPL38 (110624), ENSSSCG00000046071 (566256)*
	ALGA0080239	14	98332707	2.77E-05	Intron	*PRKG1*
	ALGA0085484	15	54398519	2.28E-05	Intron	*GSR*
	DRGA0016186	16	44656503	2.67E-07	Intergenic	*SREK1 (36409), ENSSSCG00000063417 (316356)*
ADG (g)	ALGA0006623	1	160347188	1.60E-16	5_prime_UTR	*ENSSSCG00000048538*
	ASGA0004992	1	160210902	1.70E-11	Intergenic	*CDH20 (293757), ENSSSCG00000048538 (130158)*
	ASGA0005017	1	161540913	3.45E-09	Intron	*CCBE1*
	ALGA0006690	1	162147201	4.91E-08	Intergenic	*MALT1 (2321), ALPK2 (28372)*
	H3GA0003149	1	162192627	5.04E-08	Intron	*ALPK2*
	ASGA0005021	1	161657225	7.06E-07	Intron	*CPLX4*
	ALGA0112558	1	270826950	2.61E-06	Intron	*ABL1*
	MARC0037204	1	53549421	8.37E-06	Intergenic	*CEP162 (107022), TBX18 (288768)*
	ALGA0006707	1	162345362	1.34E-05	Intron	*ENSSSCG00000052156*
	ALGA0108601	1	160978649	2.34E-05	Intergenic	*MC4R (204525), ENSSSCG00000026454 (204238)*
	ALGA0006619	1	160247234	2.63E-05	Intron	*ENSSSCG00000055614*
	ASGA0004994	1	160223900	3.27E-05	Intergenic	*CDH20 (306755), ENSSSCG00000048538 (117160)*
	ALGA0006602	1	159538854	3.35E-05	Intron	*RNF152*
	ALGA0012897	2	30421810	1.08E-05	Intergenic	*FSHB (22536), KCNA4 (183754)*
	DRGA0004818	4	59203000	1.97E-05	Intergenic	*PKIA (1225426), PEX2 (49337)*
	ALGA0105098	6	69291261	4.60E-10	Intron	*ENSSSCG00000033497*
	H3GA0023150	7	113231192	1.02E-05	Intron	*CATSPERB*
	ALGA0047819	8	43646405	1.16E-06	Intron	*CPE*
	ALGA0113142	10	48069639	2.94E-05	Intron	*PRPF18*
	ALGA0064467	12	7009388	5.21E-06	Intergenic	*RPL38 (110624), ENSSSCG00000046071 (566256)*
	ALGA0080239	14	98332707	7.24E-06	Intron	*PRKG1*
	ALGA0085484	15	54398519	4.08E-06	Intron	*GSR*
	DRGA0016186	16	44656503	8.14E-08	Intergenic	*SREK1 (36409), ENSSSCG00000063417 (316356)*
BF (mm)	ALGA0012897	2	30421810	5.07E-10	Intergenic	*FSHB (22536), KCNA4 (183754)*
	H3GA0013013	4	74199117	9.53E-06	Intergenic	*TOX (83966), NSMAF (37709)*
	MARC0036560	5	66103958	1.56E-11	Intron	*CCND2*
	H3GA0016584	5	66223267	3.44E-08	Intergenic	*CCND2 (108696), PARP11 (220227)*
	ASGA0026705	5	89547600	2.14E-05	Intergenic	*CRADD (67888), SOCS2 (33490)*
	H3GA0017689	6	22794083	1.77E-05	Intergenic	*ENSSSCG00000044968 (1634422), CDH8 (129698)*
	H3GA0020739	7	29948363	3.25E-05	5_prime_UTR	*IP6K3*
	ASGA0038525	8	32008618	1.68E-09	Intron	*APBB2*
	ALGA0119031	9	36252818	8.07E-06	Intergenic	*SLN (10413), ENSSSCG00000058217 (17116)*
EMA (cm^2^)	ASGA0005017	1	161540913	5.26E-08	Intron	*CCBE1*
	ALGA0006623	1	160347188	1.69E-07	5_prime_UTR	*ENSSSCG00000048538*
	ALGA0006690	1	162147201	3.13E-07	Intergenic	*MALT1 (2321), ALPK2 (28372)*
	H3GA0003149	1	162192627	3.59E-07	Intron	*ALPK2*
	ALGA0007330	1	187227296	3.00E-06	Intron	*ARMH4*
	ASGA0004992	1	160210902	7.09E-06	Intergenic	*CDH20 (293757), ENSSSCG00000048538 (130158)*
	ASGA0005021	1	161657225	2.17E-05	Intron	*CPLX4*
	ALGA0001723	1	22652735	2.35E-05	Intron	*ENSSSCG00000063202*
	ALGA0025513	4	65186206	3.27E-06	Intron	*NCOA2*
	ASGA0092589	6	155868122	1.89E-05	3_prime_UTR	*PLPP3*
	ASGA0032514	7	30234691	4.26E-06	Intron	*GRM4*
	ASGA0038525	8	32008618	2.25E-05	Intron	*APBB2*

SNP, single nucleotide polymorphism; SSC, *Sus scrofa* chromosome; AGE, age to 105 kg; UTR, untranslated region; ADG, average daily gain; BF, backfat thickness; EMA, eye muscle area.

1) Bold indicate p-values exceeding the Bonferroni significance threshold.

2) Gene symbols when intronic or gene symbols (distance) adjacent to the marker when intergenic.

**Table 3:** Significant gene ontology (GO) terms and Kyoto encyclopedia of genes and genomes (KEGG) pathways associated with productive traits in Yorkshire pig (p<0.05)

Gene ontology and KEGG pathway	Count	p-value	Gene
GO:0032743-positive regulation of interleukin-2 production	2	0.03	*ABL1, MALT1*
GO:0045780-positive regulation of bone resorption	2	0.02	*FSHB, MC4R*
GO:0046854-phosphatidylinositol phosphorylation	2	0.02	*IP6K3, SOCS2*
GO:0042981-regulation of apoptotic process	3	0.02	*ABL1, CRADD, MALT1*
GO:0034332-adherens junction organization	2	0.04	*CDH20, CDH8*
GO:0016339-calcium-dependent cell-cell adhesion via plasma membrane cell adhesion molecules	2	0.04	*CDH20, CDH8*
GO:0007043-cell-cell junction assembly	2	0.04	*CDH20, CDH8*
GO:0098742-cell-cell adhesion via plasma-membrane adhesion molecules	2	0.05	*ABL1, MALT1*
GO:0002020-protease binding	3	0.01	*CRADD, MALT1, CCBE1*
GO:0016342-catenin complex	2	0.04	*CDH20, CDH8*
ssc04917: Prolactin signaling pathway	2	0.03	*CCND2, SOCS2*

## SUPPLEMENTARY MATERIAL

**Supplementary Table S1**. Basic statistics for productive traits of Yorkshire pig **Supplementary Figure S1**. LD block analysis between significant markers found SSC 1 region using Haploview for (A) AGE and (B) EMA.
