# Supplementary material for: Genome-wide association study to reveal new candidate genes using single-step approaches for productive traits of Yorkshire pig in Korea
Source: Anim Biosci. 2024 Jan 20;37(3):451–60. doi: 10.5713/ab.23.0255 (PMC10915189; doi:10.5713/ab.23.0255)
Supplement: Supplementary file 1 [file ab-23-0255-Supplementary.pdf]

SUPPLEMENTARY MATERIAL

Supplemntary Table S1 Basic statistics for productive traits of Yorkshire pig.
Supplemntary Figure S1 LD block analysis between significant markers found SSC 1 region using Haploview for (A) AGE and (B) EMA. The red horizontal line indicates the Bonferroni significance threshold 1.68×10^{-6}

Table S1. Basic statistics for productive traits of Yorkshire pig.

Traits	No. of records	Mean	SD[1]	Minimum	Maximum	CV (%)[3]
AGE (days)	104,380	156.98	12.38	121.33	222.36	7.89
ADG (g)	104,380	672.98	52.38	472.21	865.41	7.78
BF (mm)	104,380	14.12	2.96	6.14	33.48	20.96
EMA (cm^2)	104,380	29.73	3.12	11.31	64.64	10.49

[1]Standard deviation; [2]coefficient of variation.

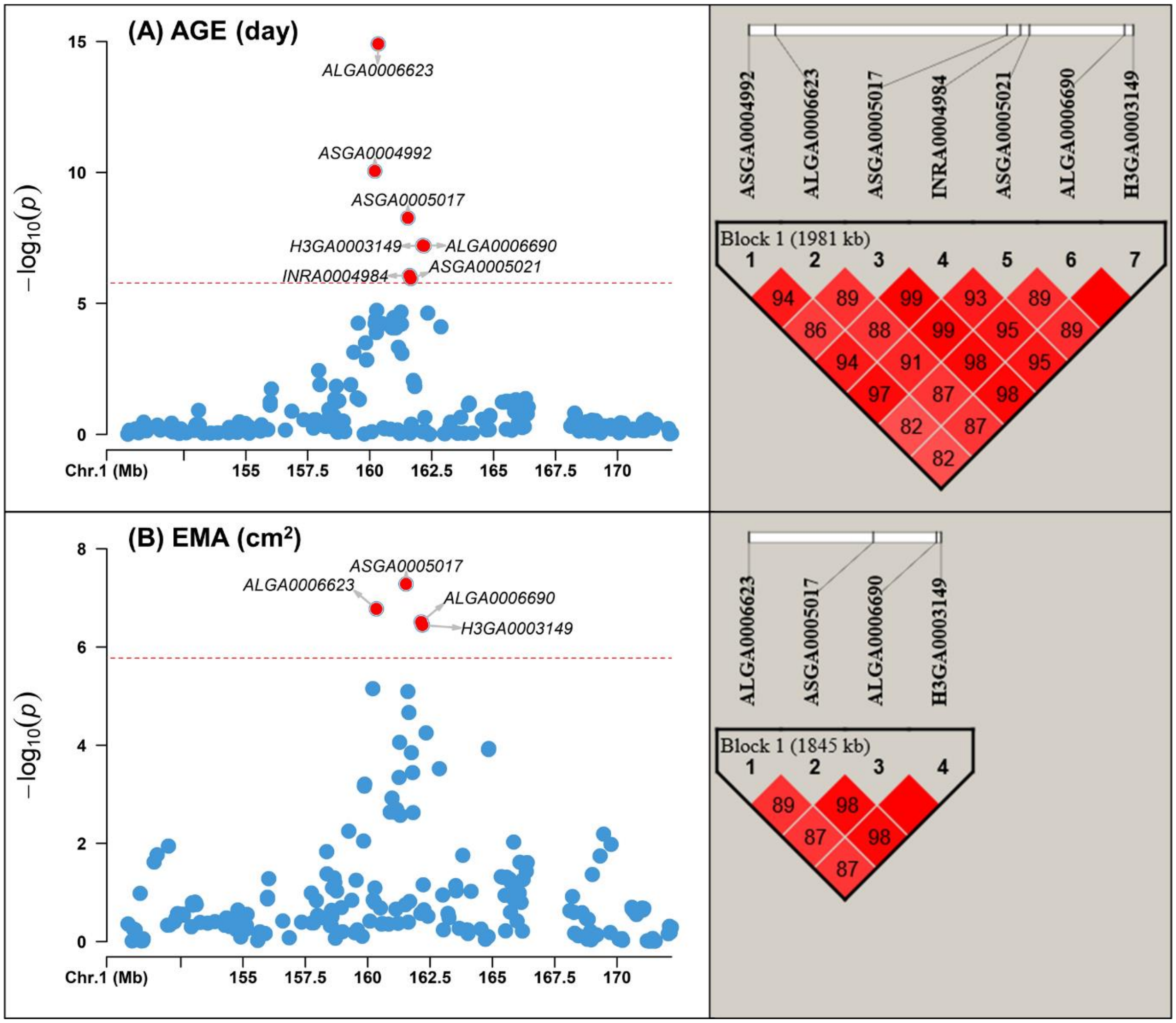

Figure S1. LD block analysis between significant markers found SSC 1 region using Haploview for (A) AGE and (B) EMA. The red horizontal line indicates the Bonferroni significance threshold 1.68×10^{-6}
